# On Prolonging Network Lifetime through Load-Similar Node Deployment in Wireless Sensor Networks

**DOI:** 10.3390/s110403527

**Published:** 2011-03-24

**Authors:** Qiao-Qin Li, Haigang Gong, Ming Liu, Mei Yang, Jun Zheng

**Affiliations:** 1 School of Computer Science and Engineering, University of Electronic Science and Technology of Chengdu, Chengdu 610054, China; E-Mails: helenli803@uestc.edu.cn (Q.-Q.L.); csmliu@uestc.edu.cn (M.L.); 2 Department of Electrical and Computer Engineering, University of Nevada, Las Vegas, NV 89154, USA; E-Mail: meiyang@egr.unlv.edu; 3 Department of Computer Science and Engineering, New Mexico Institute of Mining and Technology, Socorro, NM 87801, USA; E-Mail: zheng@cs.nmt.edu

**Keywords:** energy hole, load-similar node distribution, energy balance, network lifetime

## Abstract

This paper is focused on the study of the energy hole problem in the Progressive Multi-hop Rotational Clustered (PMRC)-structure, a highly scalable wireless sensor network (WSN) architecture. Based on an analysis on the traffic load distribution in PMRC-based WSNs, we propose a novel load-similar node distribution strategy combined with the Minimum Overlapping Layers (MOL) scheme to address the energy hole problem in PMRC-based WSNs. In this strategy, sensor nodes are deployed in the network area according to the load distribution. That is, more nodes shall be deployed in the range where the average load is higher, and then the loads among different areas in the sensor network tend to be balanced. Simulation results demonstrate that the load-similar node distribution strategy prolongs network lifetime and reduces the average packet latency in comparison with existing nonuniform node distribution and uniform node distribution strategies. Note that, besides the PMRC structure, the analysis model and the proposed load-similar node distribution strategy are also applicable to other multi-hop WSN structures.

## 1. Introduction

Due to the benefits of low cost, rapid deployment, self-organization capability and cooperative data processing, wireless sensor networks (WSNs) have been proposed as a practical solution for a wide range of applications [1], such as battlefield surveillance, habitat monitoring, intelligent agriculture, home automation, *etc.* A typical WSN is composed of a large number of sensor nodes responsible for sensing data and a sink node responsible for collecting and processing data. Since the energy supply for each sensor node is usually extremely limited, energy efficiency is the primary challenge of WSNs. Previous research works have indicated that a clustered structure [2,3] and multi-hop routing [4] are essential for better energy efficiency in large scale WSNs.

In WSNs, the data traffic follows a many-to-one communication pattern. Nodes closer to the sink tend to carry heavier traffic loads, which will deplete their energy faster [5–9]. Wadaa *et al.* [8] argued that by the time nodes closest to the sink deplete their energy, nodes farther away from the sink may still have up to 93% of their initial energy available, assuming all nodes have the same fixed transmission range and nodes are uniformly distributed in the network. In the literature, this problem is referred as the *energy hole* problem and a number of studies have been conducted to mitigate its impact on the lifetime of a WSN, including energy-efficient clustering schemes [5,7,10], analysis of the energy hole problem [11,12], nonuniform node distribution strategies [13,14], and utilization of mobile nodes [15].

In our previous work, a highly scalable network architecture, named Progressive Multi-hop Rotational Clustered (PMRC) structure [16], was proposed for the construction of large scale WSNs. Like other multi-hop structures, PMRC also suffers from the energy hole problem. This issue was alleviated to some extent by the Overlapping Layers (OL) scheme [17], which tends to balance the relay load at the cluster heads for all layers by overlapping the neighbor layers following a desired overlap range. However, due to the fixed layer boundary and range overlap, the network lifetime of OL-enabled WSN is still limited by those nodes which have exactly one candidate cluster head. To overcome this limit, the Minimum Overlapping Layers (MOL) scheme [18] was proposed to gradually increase the required minimum overlap between neighbor layers during network lifetime. As the traffic follows a many-to-one pattern, the network lifetime of MOL-enabled PMRC-based WSNs is still limited by the number of sensor nodes in the initial first layer. Unfortunately, the existing schemes for the energy hole problem in WSNs may not be suitable for MOL-enabled PMRC-based WSNs in which the layer boundary changes dynamically during network lifetime.

In this paper, we propose a load-similar node distribution strategy to address the energy hole problem of PMRC-based WSNs. First, a load analysis in the continuous space of the network is performed, which reflects the gradual change of the layer boundary in the MOL scheme. Then, based on the analysis, a load-similar node distribution strategy is proposed. Simulation results confirm the superiority of the proposed load-similar node distribution over the non-uniform node distribution [14] and uniform node distribution strategies for MOL-enabled PMRC-based WSNs.

The rest of this paper is organized as follows. Related work is reviewed in Section 2. The preliminaries, including the PMRC structure, the MOL scheme, and media access control are briefly described in Section 3. In Section 4, the traffic load in PMRC-based WSNs is analyzed and the load-similar node distribution strategy is described. Simulation results are presented in Section 5 and the paper is concluded in Section 6.

## 2. Related Works

Clustering structures have been proposed to balance energy consumption in WSNs. Soro and Heinzelman [10] proposed an Unequal Clustering Size (UCS) model to balance energy consumption of cluster heads in multi-hop WSNs. This work focuses on a heterogeneous network where cluster heads are deterministically deployed at some pre-computed locations, making it easy to control the cluster size. Chen *et al.* [17] proposed an Unequal Cluster-based Routing (UCR) protocol to partition the sensor nodes into clusters with unequal sizes; those clusters closer to the sink node shall have smaller cluster sizes, will consume less energy during the intra-cluster data processing, and preserve more energy for the inter-cluster relay traffic. They also proposed a greedy geographic and energy-aware multi-hop routing protocol for inter-cluster communications. Liu *et al*. [5] further investigated the theoretical aspects of the energy hole problem in wireless sensor networks with clustering. They proposed to employ an unequal cluster-radius and alternate between dormancy and work to mitigate the energy hole problem.

Perillo *et al.* [19] discussed the problems related to energy unbalance among sensors in many-to-one sensor networks with uniform node distribution. When all the sensor nodes have fixed transmission ranges, those nodes closer to the sink tend to deplete energy faster. On the other hand, if all the sensors transmit to the sink directly, sensors farther away from the sink will deplete energy faster than those closer to the sink. They thereby proposed a general model to study the optimal transmission range distribution to maximize the network lifetime. They concluded that varying the transmission range among nodes at different distances to the sink can alleviate the energy hole problem only to a limited extent, as energy balance can only be achieved at the expense of using energy resources of some nodes inefficiently.

Song *et al.* [20] proposed an improved corona model with levels for analyzing sensors with adjustable transmission ranges in WSNs with circular multi-hop deployment. They considered that the right transmission ranges of sensors in each corona is the decision factor for optimizing the network lifetime after nodes deployment. They also proved that searching optimal transmission ranges of sensors among all coronas is a multi-objective optimization problem, which is NP hard. Therefore, the authors proposed a centralized algorithm and a distributed algorithm for assigning the transmission ranges of sensors in each corona for different node distributions. The two algorithms can not only reduce the searching complexity but also obtain results approximated to the optimal solution.

Li and Mohapatra [11] developed a mathematical model to analyze the energy hole problem in a circular WSN and investigated the effect of several possible schemes that aim to mitigate the energy hole problem, such as deployment assistant, data compression and data aggregation. They assumed that nodes are uniformly and randomly distributed, and each node continuously generates constant bit rate data. Energy lost in data sensing, data transmission and reception is considered. The simulation results confirmed that hierarchical deployment, data aggragation and data compression can alleviate the energy hole problem, while under the same network diameter conditions, higher data rates will worsen the energy hole problem and higher node density cannot prolong the network lifetime.

Olariu and Stojmenović [12] were the first to study how to avoid the energy hole problem in WSNs. They investigated the theoretical aspects of uneven energy depletion problem in sink-based WSNs with uniform node distribution and constant data reporting. They assumed an energy consumption model governed by *E* = *d^α^* + *c*, where *d* is the transmission range and *c* is a positive constant parameter. They concluded that uneven energy depletion is intrinsic to the system and no routing strategy can avoid energy hole around the sink when *α* = 2. For larger values of *α*, the uneven energy consumption can be prevented by judicious system design and the energy consumption is suboptimally balanced across the network.

Lian *et al.* [13] proposed the SSEP-Non-uniform Sensor (SSEP-NS) distribution model and the SSEP-NS routing protocol to increase the network data capacity. The SSEP-NS network is partitioned into sub-areas and the sub-areas are further partitioned into sub-regions. The closer a sub-region is to the sink, the higher the sensor density in the sub-region. To save energy, sensor nodes work in an active-sleep model. The SSEP-NS routing protocol is used to maitain the property of uniformly distributed active sensor nodes with the minimum density at any time. In this work, only energy consumption for data transmission considerd.

Wu *et al.* [14] investigated the theoretical aspects of the nonuniform node distribution strategy used to mitigate the energy hole problem in WSNs. They concluded that it is impossible to achieve completely balanced energy depletion among all the nodes due to the intrinsic many-to-one traffic pattern in a circular multi-hop WSN. However, nearly balanced energy depletion in the network is possible by using their proposed nonuniform node distribution strategy, wherein the number of nodes grows in geometric progression from the outer coronas to the inner ones, except the outmost one. They assumed that the nodes in the network constantly report data to the sink and nodes are active all the time. Energy consumption for both data transmission and reception is considered. For their proposed nonuniform node distribution strategy, they also proposed the *q*-Switch routing algorithm, in which the relay node with the maximum residual energy among *q* relay nodes in the adjacent inner corona is selected. In their network model, the boundary between adjacent coronas is fixed and the network only lasts to the time when the first node is dead.

As introduced in Section 1, in MOL-enabled [18] PMRC-based WSNs, the layer boundary between neighbor layers is dynamically changed during network lifetime. As such, the aforementioned solutions may not be suitable for the energy hole problem in MOL-enabled PMRC-based WSNs.

## 3. Preliminaries

### 3.1. Network Model

**Figure 1.**
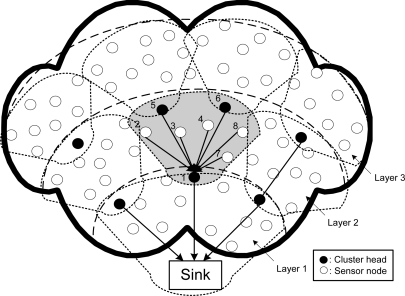
Structure of PMRC-based WSN.

Without loss of generality, we assume that all the sensor nodes are homogeneous and have the same capability. Further we assume that all sensor nodes are active in transmission and a portion of these nodes (referred as source nodes in the later text) are active in sensing data. Nodes are partitioned into layers according to their distance to the sink. A cluster is composed of sensor nodes in the same layer and a cluster head in the upstream layer. This layered model is called the PMRC-based WSN [16]. Figure 1 shows the structure of a PMRC-based network with three layers. The shadow area shows the structure of a cluster at layer 2 with node 1 (at layer 1) serving as this cluster’s cluster head.

The operation of a PMRC-based WSN is divided into rounds. Each round begins with a network formation phase when layers and clusters are formed, followed by a data gathering phase. During the network formation phase, multiple iterations are needed to discover all the sensor nodes, with one iteration dedicated for discovering the sensor nodes and forming clusters in just one layer. Each iteration begins with the broadcast of a Control Packet (CP) by the sink. In the first iteration, following the CP, sensor nodes within 1-hop distance to the sink set their layer number to 1 and select the sink as their cluster head. In the second iteration, following the CP, each node at layer 1 sends a Broadcast Packet (BP) to announce their IDs, layer number and residual energy. Upon receiving the BP, sensor nodes within a 2-hop distance to the sink respond with a Head Selection Packet (HP) to select their cluster head(s) following the cluster head selection algorithm [16]. Each selected cluster head receiving one or more HP(s) announces its cluster by broadcasting a Cluster Control Packet (CCP). This process repeats until all the nodes in the network are discovered.

**Figure 2.**
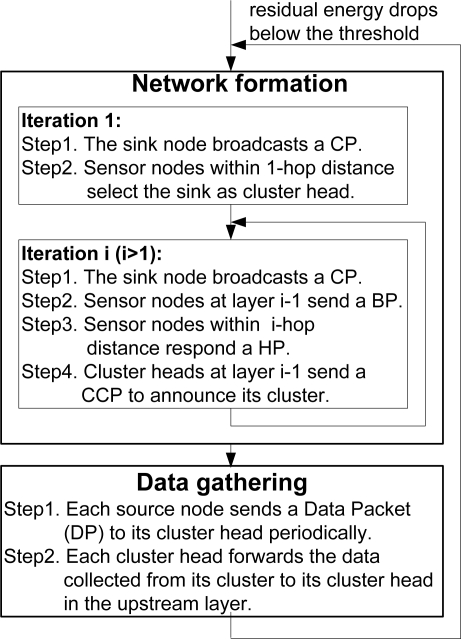
Network operation phases.

During the data gathering phase, each source node sends a Data Packet (DP) to its cluster head, either periodically or driven by an event. Each cluster head forwards the data collected from its cluster to the sink through a multi-hop path. Once the residual energy of a cluster head drops below a pre-defined threshold, it sends a beacon to announce its residual energy. Upon receiving the beacon packet, members in the cluster send a network formation request, which will be forwarded to the sink. Upon receiving a network formation request, the sink will initiate the network formation process in a new round. If there is one sensor node that cannot find any candidate cluster head during the process, the network is considered partitioned. Figure 2 illustrates the network operation phases of a PMRC-based WSN.

In the PMRC structure, two cluster head selection strategies can be used: selecting single cluster head or double cluster heads for each cluster. To further save energy, the clusters in the same layer may rotate to be active in different rounds.

### 3.2. The MOL Scheme

In the PMRC structure, the cluster heads closer to the sink node are burdened with heavy relay traffic and tend to die early, which leads to energy holes. The MOL scheme [18] is proposed to mitigate this problem and overcome the limitation imposed by the fixed layer boundary and overlap range of the OL scheme [17].

We assume that a node is eligible to be elected as a cluster head only if its residual energy is higher than some pre-defined threshold. As described in Section 3.1, when the residual energy of one cluster head falls below the threshold, a new round of network formation will be triggered. In network reformation, each node will receive the BP sent from nodes located in its inner layer and select the new cluster head. Its layer no. will be set according to the layer no. of its cluster head. As a result, some nodes may have their layer no. changed. Consequently, throughout the network lifetime, the boundary between neighbor layers is moving towards the sink as needed and the cluster size is dynamically changed.

**Figure 3.**
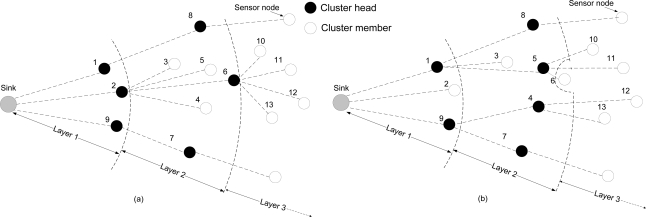
Illustration of gradually growing overlap in MOL scheme. **(a)** Initial Structure. **(b)** changed structure with dynamic layer boundary.

Figure 3 illustrates the moving of boundary between neighbor layers in the MOL scheme. Figure 3(a) shows the initial topology with node 2 serving as the cluster head for the cluster composed of nodes 3, 4, 5 and 6 at layer 2. After the residue energy of node 2 drops below the threshold, the network reformation is performed and node 1 is selected as the new head by nodes 3 and 5, as shown in Figure 3(b). Node 4, which is out of the transmission range of node 1, selects node 9 as its new cluster head. Node 6, which is originally within the transmission range of node 2, is “pushed” to layer 3 as it is out of the transmission range reachable by any node at layer 1. The result is that the boundary between layer 2 and layer 3 is moving towards the sink. Note that nodes initially belonging to layer 1 (e.g., node 2 in Figure 3) will not be pushed to layer 2 because they can still reach the sink in one hop.

The MOL scheme [18] has the following properties.: first, unlike from the OL scheme, the required minimum overlap between neighbor layers is gradually increased on demand during network lifetime. Second, the MOL scheme overcomes the limitation caused by static network topology control. As such, the MOL scheme can adapt to any randomly deployed network as long as the initial topology is connected. Third, the MOL scheme inherently helps balance the energy consumption among cluster heads within the same layer.

## 3.3. Medium Access Control

In PMRC-based WSNs, a sensor node competes the media for data transmission with all the nodes within its transmission range. A simple CSMA-based MAC scheme is implemented to avoid collisions. In this MAC scheme, when a sensor node has one or more data packets to transmit, it monitors the channel until the channel is idle before initiating the transmission. After sensing an idle channel, the sensor node generates a random backoff interval for an additional deferral time before transmission. The backoff counter is decreased by slot time as long as the channel is idle. When a transmission is detected on the channel, the backoff process is terminated and the node starts to monitor the channel again and repeat the above process. The node will transmit the data packet when the backoff counter reaches zero. The backoff time is a random number with a discrete uniform distribution between *0* and *cw*−1, where *cw* is the size of the contention window and *cw* is assumed to be a constant [21].

## 4. Load-Similar Node Distribution

### 4.1. Energy Model

In our model, each sensor node is assumed to have the same initial energy, whereas the sink has unlimited energy to consume. Assume that any sensor node is eligible to be elected as a cluster head. The energy consumed (referred as *load* in later text) by each sensor node majorly consists of three parts:

*E_t_*: the energy consumed for transmitting data generated from all sensor nodes in its cluster and the data relayed through all outer layers;
*E_r_*: the energy consumed for receiving data collected from all outer layers;
*E_c_*: the energy consumed for network formation in each round.

Following the free space channel model [2], the energy consumed for transmitting *l*-bit data over the distance of *r* is given by:

(1) $$l\left(E_{\mathrm{elec}}+{ɛr}^{2}\right),$$

where *E_elec_* and *ɛ* represent the electronic energy and amplifier energy respectively. The corresponding energy consumed in receiving *l*-bit data is *lE_elec_*. The system parameters used in this paper are set as, *E_elec_* = 50 nJ/bit, *ɛ* = 10 pJ/bit/m^2^.

The energy consumed in network formation phase is majorly composed of the energy consumed for receiving control packets, including Control Packet (CP), Header Selection Packet (HP), Broadcast Packet (BP), and Cluster Control Packet (CCP) [16]. Here, the energy consumed in sending these control packets is neglected due to the small volume of such packets. Assume the total number of layers in the network is *m*, then the energy consumed during each network formation at layer *i*, *E_ci_*, can be calculated as:

(2) $$E_{c1}=\left(2*l_{\mathrm{cp}}+l_{\mathrm{hp}}*n_{\mathrm{hp}}\right)E_{\mathrm{elec}},$$

(3) $$E_{\mathrm{ci}}=\left(l_{\mathrm{cp}}*n_{\mathrm{cp}}+l_{\mathrm{bp}}*n_{\mathrm{bp}}+l_{\mathrm{hp}}*n_{\mathrm{hp}}+l_{\mathrm{ccp}}*n_{\mathrm{ccp}}\right)E_{\mathrm{elec}},\;1<i<m,$$

(4) $$E_{\mathrm{ci}}=\left(l_{\mathrm{cp}}*n_{\mathrm{cp}}+l_{\mathrm{bp}}*n_{\mathrm{bp}}+l_{\mathrm{ccp}}*n_{\mathrm{ccp}}\right)E_{\mathrm{elec}},i=m,$$

where *l_cp_*, *l_hp_*, *l_bp_* and *l_ccp_* represent the packet length of a CP, HP, BP and CCP, respectively, while *n_cp_*, *n_hp_*, *n_bp_* and *n_ccp_* represent the respective average number of CPs, HPs, BPs and CCPs received by each node during each network formation. As we can see from Section 3.1, each node participates in the formation of its own layer and its outer layer, the energy consumed during network formation phase for each node has no relation to its distance to the sink.

### 4.2. Analysis of Load in Uniform Distribution Scheme

Assume static sensor nodes are uniformly distributed with node density *ρ* within a 2*R* × 2*R* square area, and the sink is located at the center of the area. Each source node generates and sends *λ* bits of data per unit time. The ratio of the number of source nodes to the total number of sensor nodes is *μ*.

**Figure 4.**
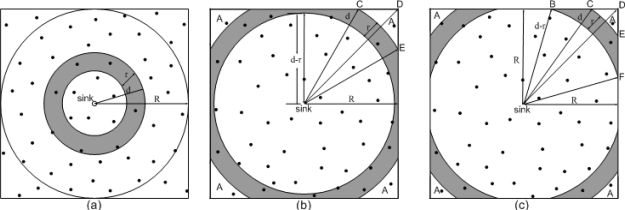
Geometry relationship of layer i. **(a)** d ≤ R. **(b)** *R* < *d* ≤ *R* + *r*. **(c)** *R* + *r*< *d* ≤ $\sqrt{2}$*R*.

Figure 4 illustrates the geometric relationship of layer *i* (with radius *r* which is equal to the transmission range) within a 2*R* × 2*R* square area. Assume the distance from the sink to the outer boundary of layer *i* is *d*. First, we deduce the average load per node at layers within the range of *r* < *d* ≤ *R*, as shown in Figure 4(a). According to the energy model, the total energy consumed for data reception and transmission in a unit time by all the sensor nodes at layer *i* (*i* > 1), *E_ri_* and *E_ti_*, are given by:

(5) $$E_{\mathrm{ri}}=E_{\mathrm{elec}}\lambda \rho \mu \left(4R^{2}-\pi d^{2}\right),\;i>1,\;r<d\leq R,$$

(6) $$E_{\mathrm{ti}}=\left(E_{\mathrm{elec}}+{ɛr}^{2}\right)\lambda \rho \mu \left(4R^{2}-\pi {\left(d-r\right)}^{2}\right),\;i>1,\;r<d\leq R.$$

Assume *N_i_* is the number of sensor nodes at layer *i*, which can be calculated by *ρπ*(*d*^2^−(*d* − *r*)^2^). Then the average load per node at layer *i* (for *i*>1 and *r*<*d*≤*R*) in a unit time is given by:

(7) $$\begin{array}{l} L_{i}=\frac{E_{\mathrm{ri}}+E_{\mathrm{ti}}}{N_{i}}+\frac{E_{\mathrm{ci}}}{T_{r}}\\ =\frac{E_{\mathrm{elec}}\lambda \rho \mu \left(4R^{2}-\pi d^{2}\right)}{\rho \pi \left(d^{2}-{\left(d-r\right)}^{2}\right)}+\frac{\left(E_{\mathrm{elec}}+{ɛr}^{2}\right)\lambda \rho \mu \left(4R^{2}-\pi {\left(d-r\right)}^{2}\right)}{\rho \pi \left(d^{2}-{\left(d-r\right)}^{2}\right)}+\frac{\left(l_{\mathrm{cp}}*n_{\mathrm{cp}}+l_{\mathrm{bp}}*n_{\mathrm{bp}}+l_{\mathrm{hp}}*n_{\mathrm{hp}}+l_{\mathrm{ccp}}*n_{\mathrm{ccp}}\right)E_{\mathrm{elec}}}{T_{r}}, \end{array}$$

where *T_r_* is the average lifetime per round, and 1/*T_r_* gives the number of network formations performed in a unit time. For *R* < *d* ≤ *R* + *r*, shown as the shaded area in Figure 4(b), the average load per node at layer *i* in a unit time can be calculated as:

(8) $$L_{i}=\frac{E_{\mathrm{ri}}+E_{\mathrm{ti}}}{N_{i}}+\frac{E_{\mathrm{ci}}}{T_{r}}=\frac{4\lambda \mu E_{\mathrm{elec}}A}{S_{1}}+\frac{\lambda \mu \left(E_{\mathrm{elec}}+{ɛr}^{2}\right)\left(4A+S_{1}\right)}{S_{1}}+\frac{\left(l_{\mathrm{cp}}*n_{\mathrm{cp}}+l_{\mathrm{bp}}*n_{\mathrm{bp}}+l_{\mathrm{hp}}*n_{\mathrm{hp}}+l_{\mathrm{ccp}}*n_{\mathrm{ccp}}\right)E_{\mathrm{elec}}}{T_{r}},$$

where *A* gives the area of each corner outside the shaded area (shape CDE in Figure 4(b)), which can be calculated by $\left(R-\sqrt{d^{2}-R^{2}}\right)R-\left(\frac{\pi }{4}-a\;\text{cos}\left(\frac{R}{d}\right)\right)d^{2}$, and *S_1_* is the area of the shaded layer which can be calculated by $4\left(R^{2}-\frac{\pi }{4}{\left(d-r\right)}^{2}-A\right)$.

For $R+r<d<\sqrt{2}R$, shown as the shaded area in Figure 4(c), the average load per node at layer *i* in a unit time can be calculated as:

(9) $$L_{i}=\frac{E_{\mathrm{ri}}+E_{\mathrm{ti}}}{N_{i}}+\frac{E_{\mathrm{ci}}}{T_{r}}=\frac{\lambda \mu E_{\mathrm{elec}}A}{A\prime -A}+\frac{\lambda \mu \left(E_{\mathrm{elec}}+{ɛr}^{2}\right)A\prime }{A\prime -A}+\frac{\left(l_{\mathrm{cp}}*n_{\mathrm{cp}}+l_{\mathrm{bp}}*n_{\mathrm{bp}}+l_{\mathrm{hp}}*n_{\mathrm{hp}}+l_{\mathrm{ccp}}*n_{\mathrm{ccp}}\right)E_{\mathrm{elec}}}{T_{r}},$$

where *A*′ gives the area of shape BCDEF, which can be calculated as $\left(R-\sqrt{{\left(d-r\right)}^{2}-R^{2}}\right)R-\left(\frac{\pi }{4}-a\left(\frac{R}{d-r}\right)\right){\left(d-r\right)}^{2}$, and the calculation of the area of each corner *A* follows that in (5-2).

For $d=\sqrt{2}R$, *i.e.*, at the outermost layer, the average load per node at layer *i* in a unit time can be calculated as:

(10) $$L_{i}=\frac{E_{\mathrm{ti}}}{N_{i}}+\frac{E_{\mathrm{ci}}}{T_{r}}=\mu \lambda \left(E_{\mathrm{elec}}+{ɛr}^{2}\right)+\frac{\left(l_{\mathrm{cp}}*n_{\mathrm{cp}}+l_{\mathrm{bp}}*n_{\mathrm{bp}}+l_{\mathrm{ccp}}*n_{\mathrm{ccp}}\right)E_{\mathrm{elec}}}{T_{r}}.$$

For sensor nodes at layer 1, they will receive and forward the data coming from outside layer 1 and also send the data generated from layer 1. Then we have:

(11) $$E_{r1}=E_{\mathrm{elec}}\lambda \rho \mu (4R^{2}-{\pi r}^{2}),$$

(12) $$E_{t1}=\left(E_{\mathrm{elec}}+{ɛr}^{2}\right)\lambda \rho \mu 4R^{2}.$$

Here, *L_1_* is obtained as:

(13) $$L_{1}=\frac{E_{r1}+E_{t1}}{N_{1}}+\frac{E_{c1}}{T_{r}}=\frac{E_{\mathrm{elec}}\mu \lambda \left(8R^{2}-{\pi r}^{2}\right)}{{\pi r}^{2}}+\frac{ɛ\mu \lambda 4R^{2}}{\pi }+\frac{E_{\mathrm{elec}}\left(2*l_{\mathrm{cp}}+l_{\mathrm{hp}}*n_{\mathrm{hp}}\right)}{T_{r}}.$$

**Figure 5.**
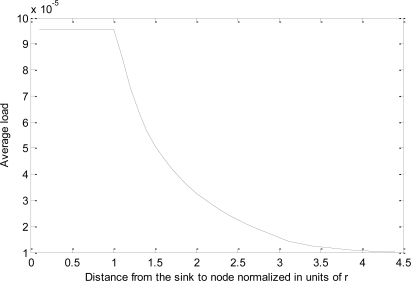
Load distribution in MOL-enabled PMRC-based WSNs.

Figure 5 depicts the average load per node *vs. d* no*rmaliz*ed in units of *r* = 40 *m* with *μ* = 20% and *λ* = 1,600 bps. The values of *l_cp_* (145 bits), *l_hp_* (169 bits), *l_bp_* (205 bits) and *l_ccp_* (259 bits) are set the same as in the simulations. Other parameters, *n_hp_* = 3.4, *n_bp_* = 6.79, *n_ccp_* = 3.4, and *T_r_* = 25 s, are estimated based on the analysis of the simulation result with uniformly distributed sensor nodes for *r* = 40 m and *ρ* = 0.0064, and these values need to be adjusted for different *r* and *ρ* values. Assuming that nodes initially at layer *i* may be pushed to layer 2*i*, then parameter *n_cp_* is calculated by (3*d*+2)/2. As shown in the figure, the average load per sensor node shows a sharp decrease with respect to the increase of the distance between the sensor node and the sink.

Agreeing with our intuition, sensor nodes at layer 1 carry the heaviest load as they have to forward all the data traffic outside layer 1. When the sensor nodes close to the sink node deplete their energy, a ring-like “hole” surrounding the sink node is created, and the sensor nodes outside the “hole” area are actually separated from the sink. As such, the network lifetime is upper-bounded by the total energy of the sensor nodes within layer 1 for the MOL-enabled PMRC-based WSN.

### 4.3. Load-Similar Node Distribution Strategy

The above analysis showed that the average load per node increases with the decrease of distance from the sink. To address the energy hole problem in the MOL-enabled PMRC-based WSN, we propose a novel load-similar node distribution strategy. The underlying principle is that if the sensor nodes are deployed in the area according to the load distribution (that is, more nodes will be deployed in the range where the average load is higher), then the load among different layers in the sensor network tends to be balanced. During the deployment of the network, the location of a node will be determined as follows:

Step 0: Compute *L*_1_, the average load per node at the outer border of layer 1;
Step 1: Randomly generate a polar coordinate, *p*(*ξ*, *θ*), centered at the sink. If *ξ*, *i.e.*, the distance between point *p* and the sink is less than the transmission range *r*, then deploy a node at location *p*, and return to Step 1 for the deployment of the next node; otherwise continue with the following steps;
Step 2: Randomly generate a value *L*′ between 0 and *L_1_*;
Step 3: Compute *L_p_*, the average load per node at location *p*. If *L_p_* > *L*′, then deploy a node at location *p*; otherwise return to Step 1 to repeat the process, until all nodes have been deployed.

**Figure 6.**
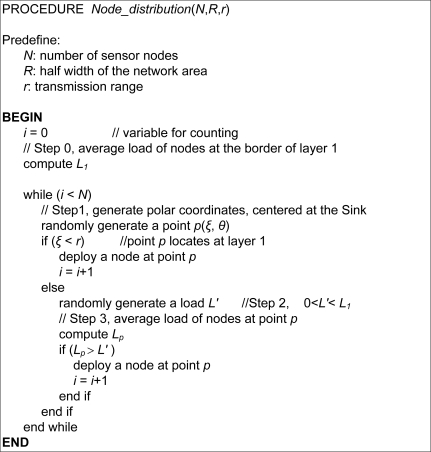
Pseudocode of load-similar node deployment.

**Figure 7.**
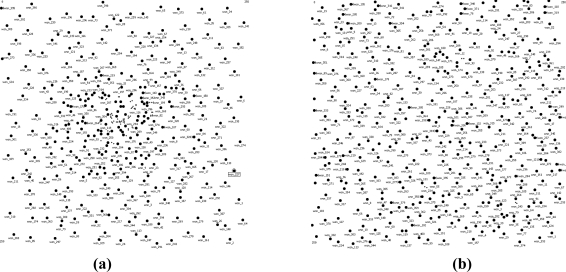
A case of node distribution (N = 400, R = 125 m, r = 40 m, sink is located at the area center). **(a)** Load-similar node distribution. **(b)** Uniform node distribution.

Assume the total number of nodes is *N*, the width of the network area is 2*R*, and the transmission range is *r*, then Figure 6 illustrates the pseudocode of load-similar node deployment. When *N* = 400, *R* = 125 m, *r* = 40 m, and the sink is located at the center of the area, an example of node deployment generated by Matlab is shown in Figure 7, where Figure 7(a) shows the load-similar node deployment and Figure 7(b) shows the uniform node deployment. It shows in Figure 7(a) that the node density is higher in the area close to the sink.

Compared with the non-uniform node distribution strategy proposed in [14], the node deployment in our strategy is very straightforward. The network formation and routing is simply based on the PMRC structure with the MOL scheme. Hence, there is no need to deploy the forwarding nodes deliberately. In addition, using the cluster structure, each node simply sends its data to its cluster head. This is a sharp contrast to the *q*-Switch routing [14], where each node needs to select one relay node with the most residual energy out of up to *q* possible forwarding nodes each time before it actually sends out its data. This puts extra requirement that the forwarding nodes periodically broadcast their residual energy, which will consume extra energy.

## 5. Performance Evaluation

To evaluate the performance of the proposed load-similar node distribution strategy for the MOL-enabled PMRC-based WSN, extensive simulations have been conducted on the WSN simulation module developed on OPNET modeler. In all simulations, we assume a 250 m × 250 m geographical area covered by a sensor network with the sink node located at the center. Table 1 lists the key parameters used in the simulations.

The following performance metrics are collected:

*Time to first node death*: in the simulation, we consider only the node death due to drained energy. Generally, this metric reflects the worst lifetime.

*Network lifetime*: it is defined as the time when the network is no longer connected or all source nodes drain out their energy.

*Number of network formations*: it is defined as the total number of network formations during network lifetime.

*Average residual energy*: it is defined as the average residual energy of all sensor nodes at their initial layer when the network lifetime ends.

*Average packet latency*: the latency of a packet is defined as the time difference between the time when the packet is generated at the source node and the time when the packet is received at the sink node.

*Data delivery ratio*: it is defined as the ratio of the number of data packets received by the sink to the total number of data packets generated in the network.

In the following, we present the simulation results of the aforementioned performance metrics for three different node distribution strategies: (1) *load-similar node distribution*, where sensor nodes are deployed following the load distribution analysis in Section 4; (2) *nonuniform node distribution*, where the number of nodes distributed in adjacent coronas *C_i_* (inner) and *C*_*i*+1_ (outer) is initially regulated as *N_i_*/*N*_*i*+1_ = *q* with a common ratio of *q* = 2; and (3) *uniform node distribution*, where sensor nodes are uniformly distributed in the area. The location of each node in these three distributions is generated using Matlab. In each simulation, a portion of sensor nodes (20% in our simulations) are selected as source nodes to generate and send data. Without loss of generality, these source nodes are randomly distributed in the network area. The results shown are the averaged results of 5 sets of source nodes.

**Figure 8.**
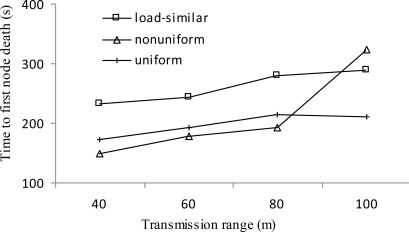
Time to first node death *vs. r*.

Figure 8 shows the time to first node death *vs.* transmission range *r*. In general, the time to first node death for all distributions shows an increasing trend with the increase of *r*. The first node death typically happens at the first layer. With the increase of *r*, the average load carried by nodes at layer 1 decreases because they will consume less energy in receiving packets as there are less nodes distributed outside layer 1. Under the same transmission range, the trend for all distributions is not consistent. That is attributed to the fact that the initial network topology also has a significant impact on the time to first node death. However, load-similar node distribution strategy achieves longer time to first node death for most cases.

**Figure 9.**
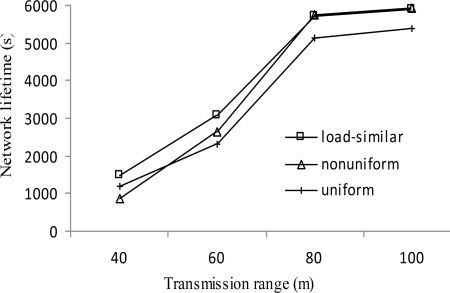
Network lifetime *vs. r*.

Figure 9 shows the network lifetime for all distributions increases monotonously with the increase of transmission range *r*. With the increase of *r*, the number of candidate cluster heads for each layer increases, which helps prolonging the network lifetime. Under the same transmission range, load-similar distribution achieves longer network lifetime than uniform node distribution (by up to 32%) for all ranges and non-uniform node distribution (by up to 73%) when *r* < 80 m. This confirms that the load-similar node distribution is more suitable for PMRC-based network than the other two distributions. When *r* ≥ 80 m, there is no significant difference between the network lifetime for load-similar distribution and non-uniform distribution as the network lifetime ends when all source nodes are exhausted.

**Figure 10.**
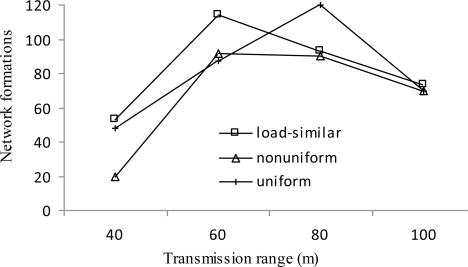
Number of network formations *vs. r*.

Figure 10 presents the number of network formations *vs.* transmission range *r*. Generally, the number of network formations shows an increasing trend followed by a decreasing trend with the increase of *r*. When the transmission range is getting larger, more candidate cluster heads are available, which leads to more rounds of network formations. For *r* ≥ 80 m, the average load at layer 1 is decreased, which leads to the longer average time per round (*i.e.*, the average time between two network formations). As a result, the number of network formations drops.

**Figure 11.**
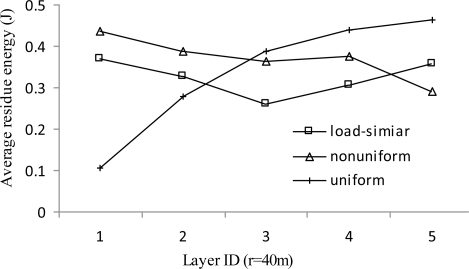
Average residue energy *vs.* layer ID (*r* = 40 *m*).

Figure 11 shows the average residual energy *vs.* layer ID when *r* = 40 m. Layers with larger layer ID are the ones farther away from the sink. The residual energy at each node is directly related to the load carried by each node. The closer to the sink, the heavier the traffic load and thus higher energy consumed for data communication. The average residual energy of the uniform distribution shows an increasing trend with the increase of layer ID, which is consistent with the load distribution analysis. The residual energy of both the load-similar distribution and nonuniform distribution is better balanced than the uniform distribution in most layers. However, the lifetime of nonuniform distribution is shorter than that of load-similar distribution and eventually more energy is wasted.

**Figure 12.**
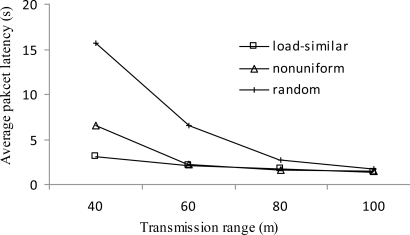
Average packet latency *vs. r*.

Figure 12 shows the average packet latency *vs.* transmission range *r*. In general, the average packet latency deceases with the increase of transmission range *r*. For the same *R*, with the increase of *r*, less layers are generated in the network; then the average number of hops traversed by each packet is decreased, which leads to a lower packet latency. Under the same transmission range, the load-similar distribution significantly reduces packet latency. This is due to the fact that during network lifetime, the number of layers in the load-similar distribution grows much slower than that in the uniform distribution and slower than that in the nonuniform distribution. That is, of the three schemes, the average number of hops traversed in the load-similar distribution is the smallest. The superiority of load-similar distribution is more evident when the transmission range is smaller.

**Figure 13.**
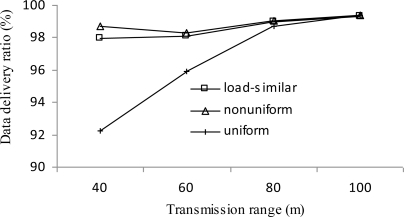
Data delivery ratio *vs. r*.

Figure 13 presents the data delivery ratio of three distribution strategies *vs.* transmission range *r*. The data delivery ratio of both the load similar distribution and nonuniform distribution is better than that of uniform distribution, especially when the transmission range is smaller. Packets that are not delivered include those lost during network reformations, and those left in the queues when network lifetime ends. The latter part dominates the overall number. As an effect of the load distribution, in uniform distribution, the traffic is more congested at those cluster heads closer to the sink than the other two distributions. The result is that more packets are left in queues in uniform distribution than the other two distributions.

## 6. Conclusions

In this paper, the energy hole problem in PMRC-based WSNs was studied. We first analyzed the traffic load distribution in PMRC-based WSNs and showed that the average load per sensor node increases as the distance from the sink decreases. Based on the analysis, we proposed a novel load-similar node distribution strategy combined with the MOL scheme to alleviate the energy hole problem in PMRC-based WSNs. Extensive simulations have been conducted to validate the analysis. The simulation results confirmed that the proposed load-similar node distribution strategy achieves good energy balance among different layers in the network and prolongs network lifetime than an existing nonuniform node distribution and uniform node distribution strategies. The superiority of the load-similar node distribution strategy is more evident when there are more layers in the network. Note that although the authors demonstrated the load-similar node distribution strategy is best suitable for PMRC-based WSNs, the analysis model and the proposed load-similar distribution strategy actually can be well applied to other multi-hop WSN structures.

## Figures and Tables

**Table 1.** Simulation parameters.

**Parameter**	**Value**
Number of nodes	400
Radio transmission range	{40, 60, 80, 100} m
Initial energy per node	0.5 J
Packet generation rate	1 *pkt/s*
Packet length	200 Bytes
Simulation time	Until network partition
